# MATE1 regulates cellular uptake and sensitivity to imatinib in CML patients

**DOI:** 10.1038/bcj.2016.79

**Published:** 2016-09-16

**Authors:** S Harrach, C Schmidt-Lauber, T Pap, H Pavenstädt, E Schlatter, E Schmidt, W E Berdel, U Schulze, B Edemir, S Jeromin, T Haferlach, G Ciarimboli, J Bertrand

**Affiliations:** 1 Institute of Experimental Musculoskeletal Medicine, University Hospital Münster, Münster, Germany; 2 Experimental Nephrology, Medizinische Klinik D, University Hospital Münster, Münster, Germany; 3 Molecular Nephrology, Department of Internal Medicine D, University Hospital Münster, Münster, Germany; 4 Department of Medicine A, University Hospital Münster, Münster, Germany; 5 Clinic and Polyclinic of Internal Medicine IV, Hematology and Oncology, University Hospital Halle, Halle (Saale), Germany; 6 MLL Munich Leukemia Laboratory, München, Germany; 7 Department of Orthopedic Surgery, Otto-von-Guericke University Magdeburg, Magdeburg, Germany

## Abstract

Although imatinib is highly effective in the treatment of chronic myeloid leukemia (CML), 25–30% patients do not respond or relapse after initial response. Imatinib uptake into targeted cells is crucial for its molecular response and clinical effectiveness. The organic cation transporter 1 (OCT1) has been proposed to be responsible for this process, but its relevance has been discussed controversially in recent times. Here we found that the multidrug and toxin extrusion protein 1 (MATE1) transports imatinib with a manifold higher affinity. MATE1 mainly mediates the cellular uptake of imatinib into targeted cells and thereby controls the intracellular effectiveness of imatinib. Importantly, MATE1 but not OCT1 expression is reduced in total bone marrow cells of imatinib-non-responding CML patients compared with imatinib-responding patients, indicating that MATE1 but not OCT1 determines the therapeutic success of imatinib. We thus propose that imatinib non-responders could be identified early before starting therapy by measuring MATE1 expression levels.

## Introduction

Although the tyrosine kinase inhibitor imatinib is highly effective in the treatment of chronic myeloid leukemia (CML), some patients do not respond to imatinib or relapse after initial response.^1, 2^ In all, 25–30% of CML patients treated with imatinib as a first-line therapy experience treatment failure leading to an increased risk of progression to accelerated or blast phase. The response rate varies within different stages of the disease.^3^ Besides other mechanisms of resistance such as mutations in the *BCR-ABL1* fusion gene,^4^ the transport of imatinib into its targeted cells has been proposed to be crucial for the clinical effectiveness.^5, 6, 7^ As an organic cation, imatinib has to be actively translocated across the cell membrane. Several clinical trials as well as *in vitro* studies investigated the role of the organic cation transporter 1 (OCT1) in this process. Although some studies suggested a critical role of OCT1 in regulating imatinib efficacy,^5, 6, 7^ other studies could not confirm these findings.^8, 9^ The role of OCT1 for imatinib uptake has mostly been inferred from inhibition studies with prazosin and amantadine. This approach has been criticized recently, as these substances do not specifically inhibit OCT1 but are potential inhibitors of other imatinib uptake pathways distinct from OCT1.^10^ Although OCT1 is generally capable to transport imatinib, it might have minor relevance for a transport under clinical conditions. Indeed, *in vitro* studies have shown a moderate affinity of OCT1 to imatinib^11, 12^ as well as low expression levels in mononuclear and CD34^+^ cells.^8, 13^ OCT1 belongs to the SLC22A family of polyspecific organic cation transporters that also includes OCT2 and OCT3, which have an overlapping spectrum of substrates.^14^ Experiments on model cells have shown that other members of this group also transport imatinib.^11, 12, 15^ Others and our group recently demonstrated that the multidrug and toxin extrusion protein 1 (MATE1), also belonging to the group of organic cation transporters, accepts imatinib as a substrate.^11, 12^

Transporters other than OCT1, especially MATE1, have not been investigated regarding their role for imatinib accumulation in targeted cells for CML treatment yet. Here we investigate transporters that might be relevant for imatinib uptake, and regarding their role in mediating cellular imatinib uptake, regulating molecular response and their correlation to the clinical therapeutic response to imatinib of CML patients.

## Materials and methods

### Cells

Peripheral blood mononuclear cells (PBMCs) were isolated from buffy coats of six healthy volunteers (German Red Cross) with a Ficoll density gradient assay (GE Healthcare, Freiburg, Germany) and used for experiments within 6 h. The ethics committee of the University of Münster approved these experiments. K562 cells were kindly provided by Prof. Müller-Tidow (University of Halle, Halle (Saale), Germany) and cultured in RPMI-1640 medium supplemented with 10% fetal bovine serum, 2 mm glutamine at 37 °C in 5% CO_2_. HEK293 cells were stably transfected with single OCT^11, 16^ and cultured as described.^11^ Overexpression of the single OCT was verified via real-time reverse transcription polymerase chain reaction (qRT-PCR)[Supplementary-material sup1].^11^

### mRNA expression analysis

RNA isolation and reverse transcription were performed with Qiagen RNeasy Midikit (Qiagen, Hilden, Germany) and Invitrogen Super Script III (Invitrogen, Carlsbad, CA, USA), respectively. For amplification of the respective transporters and the analyzed housekeeping genes, the following specific primer pairs were used: OCT1 S, 5′-CATCATAATCATGTGTGTTGGCC-3′ and OCT1 AS, 5′-CAAACAAAATGAGGGGCAAGGCTT-3′ OCT2 S, 5′-CATTGAACTAAGAAGAGAGACCG-3′ and OCT2 AS, 5′-CCACAGTGTACAATAGACTCCA-3′ OCT3 S, 5′-GACAAGAGAAGCCCCCAACCTGAT-3′ and OCT3 AS, 5′-CACTAAAGGAGAGCCAAAAATGTC-3′ MATE1 S, 5′-GCAACCACACTTGGAGTGATGG-3′ and MATE1 AS, 5′-GAGCAGAATTCCCACTCCGAG-3′ GAPDH S, 5′-CAAGCTCATTTCCTGGTATGAC-3′ GAPDH AS, 5′-GTGTGGTGGGGGACTGAGTGTGG-3′. Quantitative real-time PCR was then carried out with ABI PRISM 7900 Sequence Detection System (Applied Biosystems, Foster City, CA, USA) using SYBR Green PCR Master Mix (Fischer Scientific, Portsmouth, NH, USA).

### Affinity of imatinib to single OCT

To elucidate whether a transport is relevant under clinically used concentrations, peak plasma levels of imatinib were compared with apparent affinities of single OCT to imatinib as recommended by the International Transporter Consortium.^17^ Imatinib plasma levels and the unbound fraction were obtained from the literature.^18, 19^ The half-maximal inhibitory drug concentration (IC_50_) was taken from previously published data, where IC_50_ values were obtained by inhibition of the well-known model substrate 4-(4-(dimethylamino)styryl)-*N*-methylpyridinium (ASP^+^).^11^ Similar IC_50_ values were obtained when inhibiting the uptake of 1-methyl-4-phenylpyridinium iodide (MPP^+^) by imatinib underlining the stability of this method.^12^

### Cellular imatinib uptake and specific OCT inhibition

HEK293, PMBC or K562 cells were incubated with 5 μm imatinib (LC Laboratories, Woburn, MA, USA) for 10 min at 4 °C, where mainly passive diffusion of imatinib through the cell membrane is possible, and at 37 °C, where active transporter-mediated uptake occurs. The transport process of the investigated transporters was specifically inhibited by chemical substances at concentrations that are known to inhibit specifically the distinct transporters (Supplementary Table 1). MATE1-mediated transport was inhibited by 200 nm pyrimethamine (Sigma-Aldrich, Steinheim, Germany),^20^ whereas 80 μm MPP^+^ (Sigma-Aldrich) was used for OCT1 and OCT2 coinhibition.^14, 20, 21, 22, 23^ Cells were then lysed and intracellular imatinib accumulation was quantified with high-performance liquid chromatography with UV detector as described previously.^11^

### MATE1 knockdown and effects on BCR-ABL1 activation or cytokinesis

MATE1 knockdown in K562 cells was performed with Hyperfect transfection reagent (Qiagen) according to the manufacturer's instructions using either 40 nM MATE1 small interfering RNA (siRNA) (Sigma-Aldrich; 5′-GACAAUUUACUGUGAGUUA-3′) or control siRNA (Invitrogen; nonsilencing mock siRNA). The transfection efficiency was verified by PCR and western blot analysis for MATE1 as described earlier.^11^ To assess the influence of MATE1 expression on imatinib-dependent biologic effects, transfected K562 cells were incubated with imatinib at different concentrations (5 or 10 μM) for 10 min and the activity of BCR-ABL1 protein was then detected by western blot analysis using anti-phospho-ABL (Cell signaling, Frankfurt, Germany) and GAPDH antibody (Sigma-Aldrich). Further, we evaluated the clonal growth of K562 cells stably transduced with lentiviral vectors (plKO.1-GFP) delivering a short hairpin RNA against MATE1 (shMATE1, 5′-GACAAUUUACUGUGAGUUA-3′) or control mRNA (shscramble). Thereon, short hairpin-transduced K562 cells (3 × 10^3^ cells) were plated in 1 ml of methylcellulose medium (M3234 MethoCult; Stem Cell Technologies, Cologne, Germany) without cytokines in quadruplicates, supplemented with imatinib (0–1000 nM) and the colonies were counted on day 7.

### Patient analysis

In total, 30 patients with BCR-ABL1 major transcript (p210)-positive CML were included in this study after written informed consent was obtained (Ethik-Kommission der Bayerischen Landesärztekammer; Ethik-Kommission No. 05117). *BCR-ABL1* diagnostic procedures were performed in the Munich Leukemia Laboratory (MLL, München, Germany) and data were kindly provided by S Jeromin and T Haferlach. Upon diagnosis, MATE1-mRNA expression was quantified by qRT-PCR in total bone marrow (BM) cells as described above. All patients were treated with imatinib and MATE1 expression was compared between imatinib-responding and -refractory patients defined by major molecular response. *BCR-ABL1*/*ABL1* ⩽10% (International Scale) after 3 months of imatinib therapy was defined as optimal response, whereas poor response was defined by a *BCR-ABL1/ABL1* >1% after 6 months of therapy (warning and failure according to the ELN 2013 guidelines^24^). Detailed patient characteristics are given in Table 1.

### Statistical analysis

Data were analyzed with GraphPad Prism, V4.0 (GraphPad Software, San Diego, CA, USA). One-way analysis of variance with Tukey's *post hoc* test or unpaired Student's *t-*test were used for statistical analysis as indicated. Values are mean±s.e.m. with the following statistical significance levels: *P<0.05, **P<0.01, ***P<0.005 and NS=not significant.

## Results

**Figure 1:**
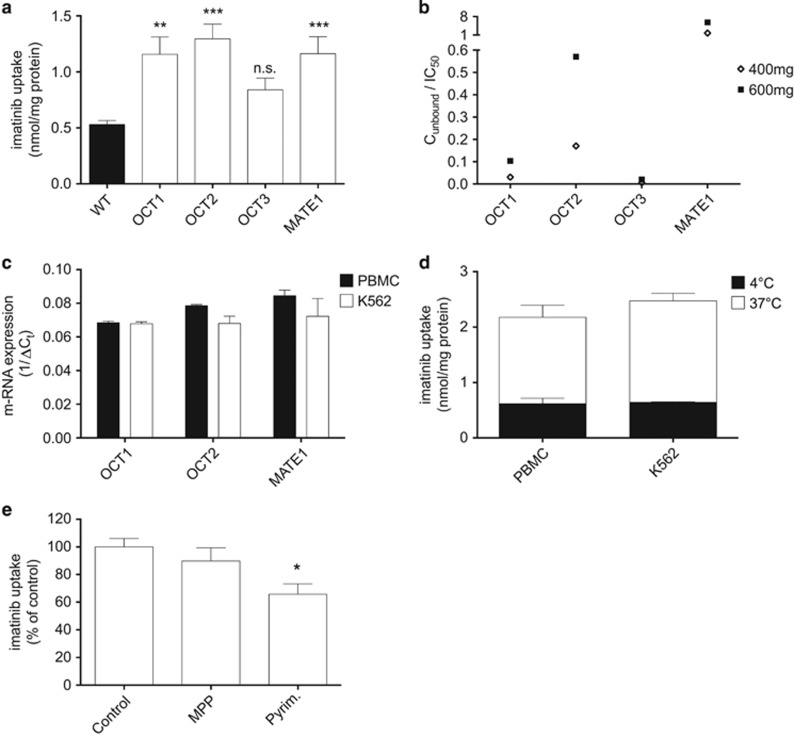
MATE1 transports imatinib with a high affinity and is the main transporter mediating imatinib uptake. (**a**) Imatinib accumulation in HEK cells overexpressing single OCTs indicates a significant transport via OCT1, OCT2 and MATE1, but not via OCT3 (*n*=4–12). Imatinib was used at 5 μM. One-way analysis of variance (ANOVA) with Tukey's *post hoc* test was used for statistical analysis. (**b**) Potential interaction of OCTs with imatinib at clinically relevant plasma levels for therapy regimens of 400 and 600 mg daily. A quotient of the unbound imatinib plasma level divided by the apparent affinities of single OCT to imatinib >0.1 indicates a possible interaction (detailed calculation see Supplementary Table 2). In contrast to OCT1 (0.03 for 400 mg and 0.1 for 600 mg imatinib daily), the quotients for MATE1 (1.7 and 5.8) and OCT2 (0.2 and 0.6) are above 0.1. (**c**) mRNA expression levels of OCT1, OCT2 and MATE1 detected by qRT-PCR in PBMCs from six healthy volunteers and in the CML cell line K562 (*n*=3–7). One-way ANOVA with Tukey's *post hoc* test was used for statistical analysis. (**d**) Temperature-dependent imatinib uptake as quantified by HPLC in PBMCs from six healthy volunteers and K562 (*n*=3–7). The significant higher uptake at 37 °C (*P*=0.0027 for PBMC; *P*=0.0001 for K562) suggests a transporter-mediated uptake. Imatinib was used at 5 μM and unpaired Student's *t*-test was used for statistical analysis. (**e**) Imatinib uptake detected by HPLC in PBMC of healthy volunteers (*n*=3–5) is reduced by specific inhibition of MATE1 with 200 nM pyrimethamine but not by OCT1 and two coinhibition with 80 μM MPP^+^. Imatinib was used at 5 μM and one-way ANOVA with Tukey's *post hoc* test was used for statistical analysis. *P<0.05; **P<0.01; ***P<0.005.

To investigate the transport capacity of OCT1 and other potential imatinib transporters, such as OCT2, OCT3 and MATE1, we measured the imatinib uptake in HEK293 cells overexpressing the respective OCTs. Imatinib uptake as detected by HPLC was significantly increased in OCT1- (1.16±0.16 nmol/mg protein; *P*=0.0013), OCT2- (1.30±0.13 nmol/mg protein; *P*=0.0001) and MATE1-overexpressing HEK cells (1.16±0.15 nmol/mg protein; *P*=0.0002), but not in OCT3-HEK cells (0.84±0.10 nmol/mg protein; *P*=0.2105) compared with wild-type HEK cells (0.53±0.03 nmol/mg protein) (Figure 1a). This indicates that OCT1, OCT2 and MATE1 but not OCT3 can generally function as imatinib transporters.

To elucidate whether this transport is relevant under clinically used concentrations, we compared imatinib plasma levels to apparent affinities of the transporters according to the recommendations of the International Transporter Consortium.^17^ Along with these guidelines, transporters with a quotient of unbound plasma drug concentration (*C*_unbound_) divided by IC_50_ >0.1 are defined as clinically relevant. Imatinib plasma levels for therapy regimens of 400 and 600 mg daily, the unbound fraction and the half-maximal inhibitory imatinib concentration to the respective OCTs (IC_50_) were obtained from the literature (Supplementary Table 2).^11, 18, 19^ For OCT1, the quotient of the unbound imatinib plasma concentration for therapy regimens of 400 or 600 mg daily divided by the apparent affinity to imatinib was 0.03 and 0.1, respectively. A relevant interaction of OCT1 with imatinib under clinically used concentration is thus unlikely. However, we found remarkably high quotients for MATE1 with 1.7 (for 400 mg imatinib daily) and 5.8 (for 600 mg imatinib daily), suggesting a possible interaction at clinically used concentrations. Quotients for OCT2 were manifold lower compared with that for MATE1, but in contrast to those of OCT1 still in the range of a potential contribution under clinically relevant concentrations (0.2 for 400 mg and 0.6 for 600 mg imatinib daily).

Besides the affinities of an OCT to imatinib, their expression level in targeted cells is important for a relevant cellular uptake of imatinib in patients. In a first step, we thus measured mRNA expression levels of the potential imatinib transporters in PBMCs of healthy volunteers as well as in the immortalized CML cell line K562, derived from a *BCR-ABL*-positive CML patient in blast crisis.^25^ In both cell types mRNA for OCT1 (1/Δ*C*_t_= 0.07 and 0.07, NS), OCT2 (0.08 and 0.07, NS), and MATE1 (0.09 and 0.07, NS) in PBMC and K562 cells, respectively, were expressed at similar levels (Figure 1c).

**Figure 2:**
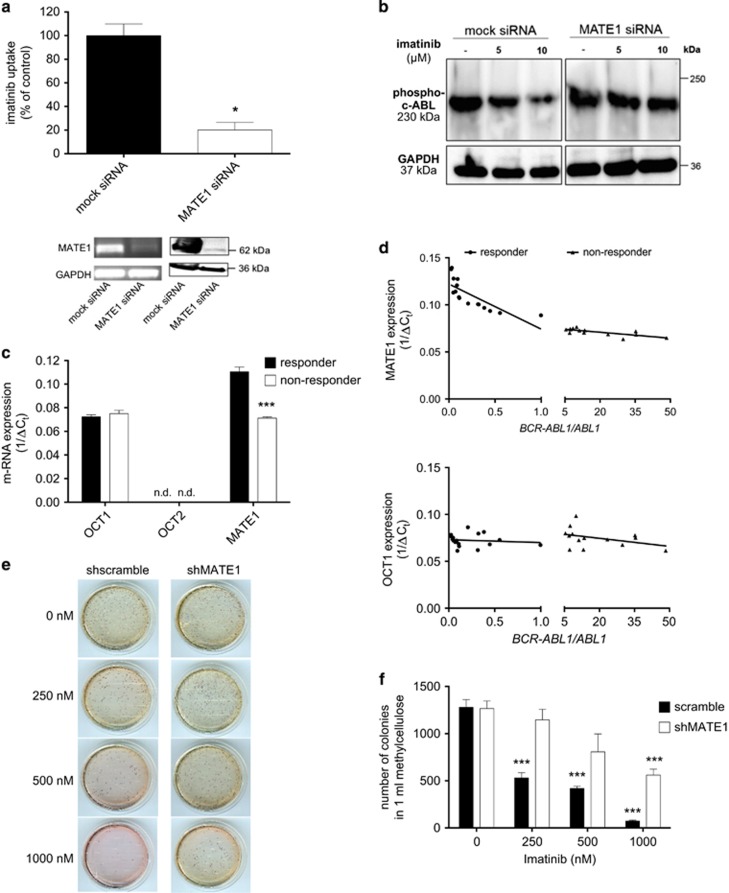
MATE1 mediates imatinib uptake in target cells and MATE1 expression levels correlate with sensitivity of CML patients to imatinib. (**a**) MATE1 knockdown in K562 cells with specific siRNA reduces the imatinib uptake compared with mock siRNA-transfected cells detected by HPLC (*n*=4). MATE1 knockdown was confirmed by RT-PCR and western blot analysis (lower panel). Imatinib was used at 5 μM and unpaired Student's *t*-test was used for statistical analysis. (**b**) Concentration-dependent effects of imatinib (5 and 10 μM) on c-ABL phosphorylation in MATE1- and mock siRNA-transfected K562 cells as quantified by western blot analysis in relation to the housekeeping gene *GAPDH*. (**c**) MATE1 and OCT1 mRNA expression in relation to the housekeeping gene. (**d**) OCT expression levels were analyzed in total BM cells of CML patients by qRT-PCR and correlated with the molecular response to imatinib treatment. MATE1 (1/Δ*C*_t_=0.11±0.04, *P*<0.0001 for responders and 1/ΔC_t_=0.07±0.001 for non-responders, *P*<0.0001) but not OCT1 (1/Δ*C*_t_=0.07±0.002 for responders and 1/Δ*C*_t_=0.08±0.003 for non-responders, *P*=0.4102) expression is lower in imatinib non-responders compared with responders (**c**) and correlates with the *BCR-ABL1/ABL1*. Thirty patients with BCR-ABL1 major transcript (p210)-positive CML were included in this study and unpaired Student's *t*-test was used for statistical analysis. (**e** and **f**) MATE1 knockdown in K562 cells with specific siRNA reduces the concentration-dependent effects of imatinib on colony growth compared with shscramble-transduced K562 cells (*n*=4). Unpaired Student's *t*-test was used for statistical analysis. *P<0.05; ***P<0.005.

To elucidate whether the imatinib uptake in these cells is mediated by transporters in general and not only due to passive diffusion through the cell membrane, we measured the uptake at 37 °C and 4 °C, where transporter-mediated uptake is strongly reduced. For PBMC and K562 cells, the uptake was significantly higher at 37 °C (PBMC: 1.56±0.22 nmol/mg protein, *P*=0.0027; K562: 1.83±0.14 nmol/mg protein, *P*=0.0001) than at 4 °C (PBMC: 0.62±0.10 nmol/mg protein; K562: 0.64±0.01 nmol/mg protein). This suggests a transporter-mediated process as the main mechanism of imatinib uptake (Figure 1d). In an attempt to identify the responsible transporters, we performed uptake experiments in the presence of specific transporter inhibitors and confirmed our finding using a gene-knockdown approach. Specific inhibitors were chosen from the literature (Supplementary Table 1).^14, 20, 21, 22, 23, 26, 27^ The selective inhibition of MATE1 with pyrimethamine significantly reduced the imatinib uptake in PBMCs by 34±8% (*P*=0.0262), whereas coinhibition of OCT1 and OCT2 with MPP^+^ had no significant effect (*P*=0.6051) (Figure 1e). Most of the studies showing that OCT1 is important for imatinib uptake and molecular response in CML patients used prazosin and amantadine as OCT1 inhibitors.^5, 28^ However, in accordance with recently published data,^10^ prazosin and amantadine are not specific inhibitors for OCT1 but have similar or even higher affinities to MATE1 than to OCT1 (Supplementary Table 1). The experiments with pyrimethamine show that this substance at the concentration used did not inhibit the imatinib uptake completely. We did not increase the pyrimethamine concentration to reach a complete suppression of imatinib cellular accumulation, to avoid additional inhibition of other OCT. To unequivocally confirm the role of MATE1 in the process of imatinib uptake, we performed a gene knockdown of *MATE1* in K562 cells using specific siRNA. The knockdown of *MATE1*, as confirmed by western blot analysis, led to an almost complete inhibition of the imatinib uptake compared with mock siRNA-transfected cells (−80±12%, *P*=0.0005) (Figure 2a). Consequently, we found that imatinib-induced inhibition of c-ABL phosphorylation was remarkably reduced in siMATE1-transfected cells compared with mock-transfected cells (Figure 2b). These results clearly show that MATE1 is the major transporter for imatinib uptake in K562 cells and is probably crucial for its intracellular therapeutic effects.

To further prove that MATE1 has an important role in regulating imatinib sensitivity in CML patients, we analyzed the transporter profile of total BM samples obtained from CML patients by qRT-PCR and correlated the expression levels with the major molecular response to imatinib treatment (Figures 2c and d). As some patients fail to respond to imatinib therapy because of *BCR-ABL1* mutations,^4^ patients with these mutations were excluded from the analysis. OCT2 mRNA was not expressed at detectable levels in the BM of all patients. The expression of mRNA for OCT1 was low (1/Δ*C*_t_=0.07±0.002 and 1/Δ*C*_t_=0.08±0.003 for imatinib responders and non-responders, respectively) and did not differ significantly between responders and non-responders.

However, MATE1-mRNA was expressed at higher levels and furthermore significantly decreased in imatinib non-responders (1/Δ*C*_t_=0.07±0.001) compared with imatinib responders (1/Δ*C*_t_=0.11±0.04, *P*<0.0001) (Figure 2c). We found a correlation (*r*^2^=0.60 and *r*^2^=0.57 for responder and non-responder, respectively) between MATE1 transporter mRNA expression and clinical response to imatinib treatment, suggesting that MATE1 levels might enable to predict whether CML patients are likely to respond to imatinib therapy (Figure 2d). In contrast, we did not find a correlation (*r*^2^=0.01 and *r*^2^=0.15 for responder and non-responder, respectively) between OCT1 transporter expression and major molecular response (Figure 2d).

To underline the hypothesis that MATE1 expression is crucial for the response to imatinib, we examined the role of MATE1 for the reductive effect of imatinib on colony formation. Therapeutically used imatinib concentrations reduced colony growth in shscramble-transduced K562 cells concentration dependently. Imatinib (250 nm) led to a colony reduction of 58% (1280 colonies without and 532 colonies with 250 nM imatinib) and 1 μm nearly totally inhibited colony growth (74 colonies, reduction of 94%). In contrast, the effects of imatinib were reduced when MATE1 was knocked down. Thus, there was no significant reduction of colony growth by imatinib in shMATE1-transduced K562 cells until a concentration of 1 μm was used (1266 colonies without and 560 colonies with 1 μm imatinib, reduction of 56% Figures 2e and f).

## Discussion

Besides other mechanisms of resistance, the transport of imatinib into target cells was proposed to regulate the effectiveness of imatinib treatment. The role of OCT1 in this process has been discussed controversially.^5, 8, 9, 28^ Most studies showing that OCT1 mediates the cellular uptake and regulates the response to imatinib rely on inhibition experiments with prazosin or amantadine.^5, 6, 7, 28^ This approach has been criticized recently and might explain the dichotomic results as both, the inhibition with prazosin and amantadine, are not specific for OCT1.^10^ We here show that although OCT1 can potentially transport imatinib, the affinity to imatinib is too low to allow a significant interaction at therapeutically reached plasma levels. Indeed, inhibition of OCT1 and OCT2, which we also identified as a potential imatinib transporter, by MPP^+^ had no significant effect of the uptake in PMBC. In contrast, MATE1 transports imatinib with a manifold higher affinity as shown in model experiments permitting an uptake at clinically observed plasma levels. Indeed, prazosin and amantadine have similar or even higher inhibitory affinities to MATE1 than to OCT1.^21, 22, 26, 27^ The observed inhibitory effects of prazosin and amantadine on imatinib uptake and its effectiveness might thus be rather because of MATE1 than OCT1 inhibition.

Specific inhibition of single OCT is difficult as these transporters share various substrates.^14^ To further prove the role of MATE1 for the cellular uptake and effectiveness of imatinib, we thus confirm the observed results of our model experiments with both specific inhibition of MATE1 by pyrimethamine and a MATE1 gene knockdown. Pyrimethamine was used at low concentrations, which significantly but not completely reduced imatinib uptake. However, we did not increase the pyrimethamine concentration to reach a total inhibition of MATE1 to guarantee specificity. This may explain the different degree of reduction in inhibition and knockdown experiment.

The importance of MATE1-mediated imatinib uptake for therapeutic effects is supported by our transduction experiments, where MATE1 knockdown markedly reduced the effects of imatinib on c-ABL phosphorylation and colony formation. Both effects are essential in the pathogenesis of CML, and c-ABL phosphorylation has been shown to be a good predictor for the molecular response of CML patients to imatinib.^29^

Deep and early molecular response is suggested to be predictive of improved long-term outcomes in CML therapy. We therefore correlated MATE1-mRNA expression levels in CML patients with the molecular response to elucidate whether MATE1 can influence the response of CML patients to imatinib. There was a significant reduction of MATE1-mRNA expression in patients who did not respond to imatinib and a strong correlation of MATE1-mRNA levels in total BM cells with the molecular response of CML patients. Interestingly, this correlation was not observed for OCT1. Measuring MATE1-mRNA levels might therefore enable to predict whether CML patients are likely to respond to imatinib therapy. Clinical trials showed evidence that dasatinib and nilotinib generate faster and deeper short-term responses in first-line CML therapy compared with imatinib.^30, 31^ However, imatinib is the most cost-effective tyrosine kinase inhibitor therapy for CML and, in contrast to dasatinib and nilotinib, is associated with less severe side effects. Screening tools such as a MATE1 expression analysis or detection of MATE1 mutations, which have been shown to rigorously change the functionality of MATE1,^32^ might thus help to individually choose the best, safest and economically reasonable therapy.

In conclusion, we demonstrate that MATE1 is the major transporter for the cellular uptake of imatinib and crucial for the therapeutic success in CML patients. We suggest that the detailed analysis of MATE1 expression levels and mutations could be a predictor for the response to imatinib therapy.

## Figures and Tables

**Table 1:** Patient characteristics

*Pat. no.*	*Response*^a^	*WBC*^b^*/μl*	*Hb (g/dl)*	*PLT*^c^*/μl*	*Material*	*Ratio*^d^ *Month 3*	*Ratio Month 6*	*Ratio 12 months*	*Gender*^e^	*Age (years)*
1	Good	13 000	13.9	442 000	BM	0.213	0.001		1	28.9
2	Good	7,200	13.7	1 130 000	BM	0.399	0.316		2	76.6
3	Good	57 700	11.6	515 000	BM	0.742	0.055		1	52.8
4	Good	23 900	15	649 000	BM	0.15	0.05	0.004	2	64.8
5	Good	132 500	10	689 000	BM	0.739	0.383		2	57.1
6	Good	80 400	12	212 000	BM	0.109		0.000	1	47.6
7	Good	49 000	14	173 000	BM	0.322		0.083	2	56.8
8	Good	28 360	13.7	665 000	BM	0.1	0.025	0.014	1	77.2
9	Good	27 500	8.8	1 710 000	BM	0.361	0.008	0.035	1	40.9
10	Good				BM	0.112		0.009	1	26.3
11	Good	49 020	11.6	335 000	BM	0.915		0.003	1	38.8
12	Good	35 600	9.2	169000	BM	0.318	0.554	0.001	1	73.2
13	Good	13 700	10.8	639 000	BM	0.02	0.031	0.102	1	58.9
14	Good				BM	0.351	0.094	0.050	1	75.4
15	Good				BM	0.446			1	57.2
16	Poor	60 000	17	101 000	BM		7.25		2	43.2
17	Poor		10.5	580 000	BM	47.411	35.246		2	46
18	Poor	344 800	11.9	300 000	BM		48.413		1	74.4
19	Poor	126 000	12.9	539 000	BM		13.157	2.370	2	35.8
20	Poor	196 900	7.8	192 000	BM		7.035	6.849	2	60.6
21	Poor	108 000	11.6	744 000	BM		8.448		1	54.5
22	Poor	526 400	7.3	430 000	BM		23.422	6.095	2	52.1
23	Poor	157 200	15	352 000	BM	4.556	36.021	0.448	2	62.4
24	Poor	160 710	7.3	74 000	BM		13.473		2	46.5
25	Poor	186.000	12.4	843 000	BM		9.968	10.410	1	58.8
26	Poor	249 000	11.2	540 000	BM	45.473	35.46		1	70.8
27	Poor				BM		5.45		2	38.3
28	Poor	128 400	10.2	621 000	BM		11.172	2.819	1	69.9
29	Poor	51 200	9	891 000	BM		7.446		2	64
30	Poor	26 700	10.8	1 200 000	BM		29.86	0.755	1	79.5

Abbreviations: BM, bone marrow; IS, International Scale; PLT, platelets; WBC, white blood cell count in peripheral blood (cells/μl).

a Response to imatinib therapy: good=3 months after the start of imatinib therapy *BCR-ABL1/ABL1* ⩽10% poor=6 months after the start of imatinib therapy *BCR-ABL1/ABL1* >1%.

b WBC in peripheral blood (cells per μl).

c PLT per μl peripheral blood.

d *BCR-ABL1/ABL1*—ratio according to IS in %.

e 1=female; 2=male.
