# Supplementary material for: MATE1 regulates cellular uptake and sensitivity to imatinib in CML patients
Source: Blood Cancer J. 2016 Sep 16;6(9):e470–. doi: 10.1038/bcj.2016.79 (PMC5056971; doi:10.1038/bcj.2016.79)
Supplement: Supplementary Table 1 [file bcj201679x1.docx]

**Supplementary Table 1**

|  | **half-maximal inhibitory drug concentration** | | |  |
| --- | --- | --- | --- | --- |
| **drug** | **of organic cation transporters［𝜇M］** | | | **References** |
|  | **OCT1** | **OCT2** | **MATE1** |  |
| **prazosin** | 1.80 | 13.60 | 1.60 | [21, 22] |
| **amantadine** | 236.00 | 19.70 | 20.00 | [26, 27] |
| **MPP^+^** | 23.90 | 65.40 | > 100.0 | [14, 23] |
| **pyrimethamine** | 3.80 | 10.00 | 0.08 | [20] |
