# Supplementary material for: MATE1 regulates cellular uptake and sensitivity to imatinib in CML patients
Source: Blood Cancer J. 2016 Sep 16;6(9):e470–. doi: 10.1038/bcj.2016.79 (PMC5056971; doi:10.1038/bcj.2016.79)
Supplement: Supplementary Table 2 [file bcj201679x2.docx]

**Supplementary Table 2**

|  | **OCT1** | **OCT2** | **OCT3** | **MATE1** | **References** |
| --- | --- | --- | --- | --- | --- |
| imatinib peak plasma levels for 400 mg daily [𝜇M] | 4.09 | 4.09 | 4.09 | 4.09 | [18] |
| imatinib peak plasma levels for 600 mg daily [𝜇M] | 13.70 | 13.70 | 13.70 | 13.70 | [18] |
| imatinib unbound plasma fraction  [%] | 5.00 | 5.00 | 5.00 | 5.00 | [19] |
| imatinib unbound plasma concentration for 400mg daily | 0.20 | 0.20 | 0.20 | 0.20 |  |
| C (unbound 400)  [𝜇M] |  |  |  |  |  |
| imatinib unbound plasma concentration for 600mg daily | 0.68 | 0.68 | 0.68 | 0.68 |  |
| C (unbound 600)  [𝜇M] |  |  |  |  |  |
| half-maximal inhibitory imatinib concentration | 6.60 | 1.20 | 34.00 | 0.12 | [11] |
| (IC_50_) [𝜇M] |  |  |  |  |  |
| C (unbound 400) / IC_50_ | 0.03 | 0.17 | 0.01 | 1.73 |  |
| C (unbound 600) / IC_50_ | 0.10 | 0.57 | 0.02 | 5.80 |  |
